# Predictive value of postoperative C-reactive protein-to-albumin ratio for severe complications following radical gastrectomy in gastric cancer patients

**DOI:** 10.3389/fmed.2026.1789300

**Published:** 2026-04-30

**Authors:** Shoukun Chen, Yueyang Huang, Jian Niu, Pengbo Zhang

**Affiliations:** 1 Department of General Surgery, Affiliated Hospital of Xuzhou Medical University, Xuzhou, Jiangsu, China; 2 Department of Laboratory Medicine, Xuzhou Central Hospital, Xuzhou, Jiangsu, China

**Keywords:** albumin, complication, C-reactive protein/albumin ratio, gastric cancer, radical gastrectomy

## Abstract

**Aim:**

To assess the prognostic significance of the C-reactive protein-to-albumin ratio (CAR) measured on the third postoperative day (POD 3) for risk stratification of severe complications after radical gastrectomy in gastric cancer (GC) patients.

**Methods:**

This retrospective analysis examined clinical data from 200 GC patients who underwent radical gastrectomy between January 2023 and December 2024. The patients were divided into a severe complication group (Clavien-Dindo grade ≥III, *n* = 18) and a control group (*n* = 182). Albumin (ALB), C-reactive protein (CRP), as well as CAR levels were compared between the two groups at various time points. Receiver operating characteristic (ROC) curve analysis as well as multivariate logistic regression were utilized to evaluate the predictive ability of CAR and to determine independent risk factors for severe postoperative complications. Based on the critical CAR value, patients were further categorized into high and low CAR groups, followed by analyzing the incidence of mild (Clavien-Dindo grade I or II) and severe (Clavien-Dindo grade III or higher) complications, as well as the length of postoperative hospital stay.

**Results:**

On POD 3, the complication group exhibited significantly lower ALB levels as well as higher CRP and CAR levels relative to the control group (*P* < 0.01). Multivariate analysis showed that an open surgical incision, operation time of 270 minutes or longer, POD 3 CRP levels of 98.5 mg/L or higher, and POD 3 CAR levels of 2.85 or higher were associated with severe complications (all *P* < 0.05). However, because CAR is mathematically derived from CRP and albumin, the apparent independent effects of CRP and CAR should be interpreted with caution. The ROC curve for POD 3 CAR revealed an area under the curve (AUC) of 0.769, with a sensitivity of 74.5% as well as a specificity of 74.0%. Patients with a high CAR (≥2.85) experienced significantly higher rates of mild (31.58% vs. 8.06%, *P* < 0.001) as well as severe complications (18.42% vs. 3.23%, *P* < 0.001), as well as a longer postoperative hospital stay (*P* = 0.011).

**Conclusion:**

The CAR on postoperative day 3 may serve as an accessible biomarker associated with severe complications after radical gastrectomy in GC patients. However, given the limited number of severe events and the lack of internal or external validation, its discriminatory performance and clinical applicability should be interpreted cautiously and require confirmation in larger prospective studies.

## Introduction

Gastric cancer (GC) belongs to a substantial global health challenge, being one of the most frequently occurring and deadly malignancies within the digestive system (1). Recent global epidemiological statistics indicate that GC holds the position of the fifth most commonly diagnosed cancer as well as ranks fourth in cancer-linked deaths, trailing only lung, colorectal, and liver cancers (2). For patients with resectable GC, radical gastrectomy with lymphadenectomy represents the cornerstone of curative-intent treatment (3). Although there have been advancements in surgical techniques and perioperative management, postoperative complications remain a significant obstacle, hindering recovery, escalating healthcare expenditures, and compromising long-term oncological prognosis (4). Therefore, the early postoperative identification of patients at increased risk for severe complications is crucial for implementing timely interventions and improving overall prognosis.

Accumulating evidence highlights the pivotal role of systemic inflammation in cancer progression and postoperative recovery (5). Inflammatory biomarkers, containing C-reactive protein (CRP), neutrophil-to-lymphocyte ratio (NLR), as well as platelet-to-lymphocyte ratio (PLR), have been explored for their potential in predicting surgical outcomes (6). Among these, CRP—a well-established acute-phase reactant—reflects the magnitude of surgical stress and inflammatory response (7), while serum albumin (ALB) serves as a critical indicator of nutritional status (8). The integration of both parameters into the CRP-to-albumin ratio (CAR) offers a composite indicator that concurrently captures inflammatory activation and nutritional depletion, potentially providing superior prognostic insight compared to either parameter alone (9).

Although previous literatures have suggested the utility of preoperative CAR in the prediction of prognosis in multiple malignancies, including intrahepatic cholangiocarcinoma, breast cancer and thoracic esophageal cancer (10–12), its role in the early postoperative settin–particularly in the early postoperative setting-particularly in risk stratification for severe complications after GC surgery—remains underexplored.

Therefore, this research was designed to assess the predictive value of postoperative CAR, specifically measured on postoperative day 3 (POD 3), for the development of severe complications following radical gastrectomy in GC patients. By addressing this gap, we seek to provide a practical, readily available biomarker that can enhance early risk stratification and support personalized perioperative management.

## Methods

### Study design

This study adopted a retrospective design. We enrolled 200 GC patients who underwent radical gastrectomy at our hospital between January 2023 and December 2024, including 139 males and 61 females, with a mean age of (63.63 ± 10.56) years. The study protocol received approval from the hospital's ethics committee, and informed consent was obtained from all patients and their families prior to participation.

Inclusion criteria: (1) The diagnosis of GC was confirmed by postoperative pathological examination; (2) Patients adopted radical gastrectomy procedures, which included D2 lymph node dissection. These surgical approaches encompassed proximal gastrectomy, distal gastrectomy, and total gastrectomy; (3) The GC lesion had not shown any distant metastasis; (4) Complete blood CRP and ALB test results were obtained on the third day after surgery for all patients.

Exclusion criteria: (1) The patient had been diagnosed with diseases affecting peripheral blood ALB, such as liver, kidney and blood system disorders; (2) Intraoperative infusion of human ALB; (3) Combined organ resection.

### Collection of clinical data

The clinical data encompassed a range of key parameters, including the patient's age, gender, history of hypertension, history of diabetes, levels of ALB, CRP and CAR before the operation and on the 1st and 3rd days after the operation, type of incision, type of resection, operation time, intraoperative blood loss, T stage, degree of differentiation, and the maximum diameter of the tumor.

### Postoperative complication judgment criteria

Early postoperative complications are defined as those that occur within the hospital or within 30 days after the surgery. The classification of all complications adheres to the Clavien-Dindo grading system. Specifically, complications graded as I or II are categorized as mild, whereas those graded as III or higher are considered severe. Clinical tumor staging is based on the criteria outlined in the 8th edition of the AJCC/UICC Cancer Staging Manual.

Patients were categorized into the complication group, comprising individuals who experienced severe complications (grade III or above), and the control group, consisting of those without such severe complications. Subsequently, statistical analyses were performed to identify and examine the risk factors associated with the onset of severe postoperative complications.

## Statistical analysis

Statistical analyses were implemented utilizing SPSS 26.0 software. For measurement data that followed a normal distribution, the mean ± standard deviation was utilized for descriptive purposes, and comparisons were implemented utilizing the two-independent-sample *t*-test. Categorical data were presented as counts (*n*) and percentages (%), with differences between the two groups evaluated through either the χ^2^ test or Fisher's exact test, depending on the circumstances. For multivariate analysis, logistic regression was applied. Variables entering the multivariable model were analyzed as categorical variables according to the groupings shown in Table 2; specifically, POD 3 CRP was entered as < 98.5 vs. ≥98.5 mg/L, and POD 3 CAR was entered as < 2.85 vs. ≥2.85. Additionally, the Receiver Operating Characteristic (ROC) curve was plotted for assessing the predictive capacity of the CAR for severe postoperative complications. A *P*-value of less than 0.05 was deemed statistically significant. Because the cutoff value of POD 3 CAR was derived and evaluated within the same cohort, and no internal or external validation was available, the ROC findings were interpreted as exploratory. To further assess the stability of the multivariable logistic regression model, internal validation was performed using bootstrapping with 1,000 resamples. Additionally, sensitivity analyses were conducted by sequentially excluding patients with extreme CAR values and those with major protocol deviations to evaluate the robustness of the association between POD 3 CAR and severe complications.

## Results

### Incidence of postoperative complications

Out of the 200 patients in the study, 52 individuals (26.00%) encountered early postoperative complications. Specifically, 34 patients (17.00%) suffered from complications classified as Clavien-dindo grade I or II. On the other hand, 18 patients (9.00%) experienced complications of Clavien-dindo grade III or higher. The complications included anastomotic fistula (3 cases), incision dehiscence (3 cases), adhesive intestinal obstruction (2 cases), lymphatic leakage (2 cases), and intra-abdominal hemorrhage (1 case) (as detailed in Table 1).

**Table 1:** Incidence of early postoperative complications among 200 GC patients after radical gastrectomy.

Postoperative complication	*n* (%)
Clavien-dindo grade I or II complications	34 (17.00)
Clavien-dindo grade III or above complications	18 (9.00)
Anastomotic fistula	5 (2.50)
Incision dehiscence	5 (2.50)
Adhesive intestinal obstruction	3 (1.50)
Lymphatic leakage	3 (1.50)
Intra-abdominal hemorrhage	2 (1.00)
Total	52 (26.00)

### CRP, ALB and CAR between the two groups

**Figure 1:**
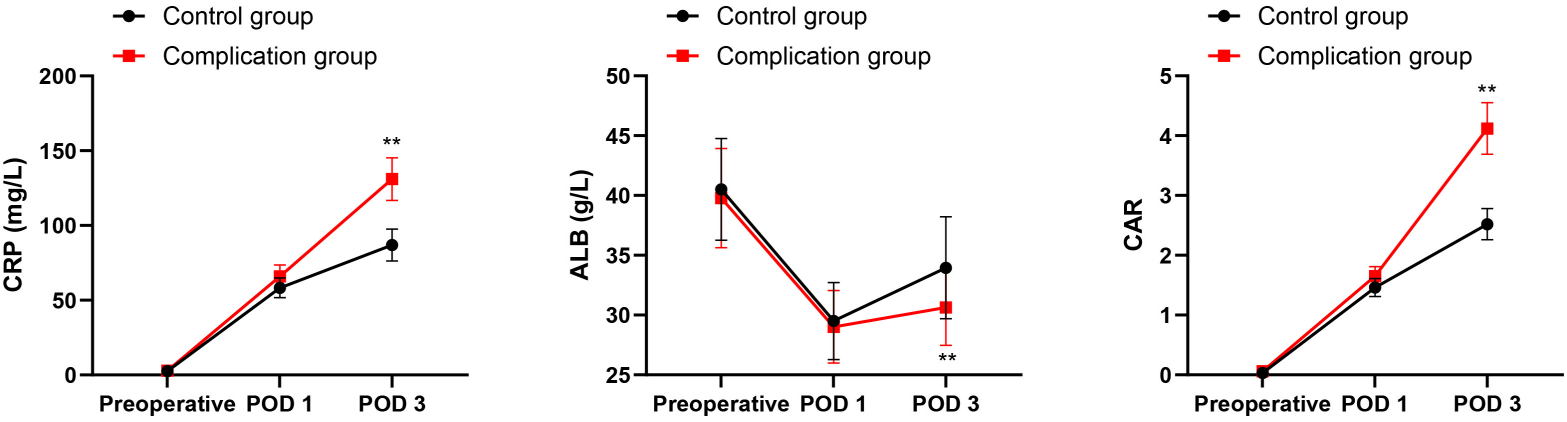
CRP, ALB and CAR between the two groups. **P < 0.01, compared with the control group.

The postoperative ALB levels in both groups showed a downward trend, while the CRP and CAR showed an upward trend. Prior to the operation and on the first postoperative day, no statistically significant disparities were reported in CRP, ALB, or CAR levels between both groups (*P* > 0.05). On POD 3, the complication group displayed lower ALB levels compared to the control group, while their CRP and CAR levels were elevated (*P* < 0.01, as illustrated in Figure 1).

### General information between the two groups

The proportions of patients in the Complication group who aged ≥ 60 years old, who underwent open incisions, whose operation duration ≥ 270 min, whose tumor maximum diameter ≥5 cm, CRP on POD 3 ≥98.5 mg/L and CAR on POD 3 ≥2.85 were all higher than those in the Control group (*P* < 0.05, Table 2).

**Table 2:** Comparison of general information between the two groups.

Characteristic	Control group (*n* = 182)	Complication group (*n* = 18)	χ^2^/Fisher's exact test	*P*
Gender				
Male	126 (69.23)	13 (72.22)	0.069	0.792
Female	56 (30.77)	5 (27.78)		
Age				
< 60 years	66 (36.26)	2 (11.11)	4.618	0.031
≥60 years	116 (63.74)	16 (88.89)		
**Hypertension**	50 (27.47)	4 (22.22)	0.040	0.841
**Diabetes mellitus**	20 (10.99)	2 (11.11)	0.143	0.704
Incision type				
Laparoscope	76 (41.76)	3 (16.67)	4.315	0.037
Open	106 (58.24)	15 (83.33)		
Type of resection				
Total gastrectomy	106 (58.24)	12 (66.67)	0.705	0.702
Proximal gastrectomy	20 (10.99)	1 (5.55)		
Distal gastrectomy	56 (30.77)	5 (27.78)		
Operation time				
< 270 min	131 (71.98)	8 (44.44)	5.858	0.015
≥270 min	51 (28.02)	10 (55.56)		
Intraoperative blood loss				
< 200 ml	40 (21.98)	5 (27.78)	0.070	0.790
≥200 ml	142 (78.02)	13 (72.22)		
T stage				
1	35 (19.23)	3 (16.67)	/	0.962
2	25 (13.74)	2 (11.11)		
3	25 (13.74)	3 (16.67)		
4	97 (53.29)	10 (55.55)		
Differentiated degree				
Low	76 (41.76)	9 (50.00)	0.749	0.687
Moderate	86 (47.25)	8 (44.44)		
High	20 (10.99)	1 (5.56)		
Tumor diameter				
< 5 cm	123 (67.58)	8 (44.44)	3.880	0.048
≥5 cm	59 (32.42)	10 (55.56)		
CRP on POD 3				
< 98.5 mg/L	109 (59.89)	5 (27.78)	6.891	0.008
≥98.5 mg/L	73 (40.11)	13 (72.22)		
CAR on POD 3				
< 2.85	118 (64.84)	6 (33.33)	6.899	0.008
≥2.85	64 (35.16)	12 (66.67)		

### Multivariable logistic regression analysis of factors associated with severe postoperative complications after radical gastrectomy in GC patients

In the multivariable logistic regression analysis, all variables, including CRP on POD 3, were entered as dichotomous variables based on the cutoffs identified in univariate analysis (Table 2). Open surgery, operation duration ≥270 min, CRP on POD 3 ≥98.5 mg/L, and CAR on POD 3 ≥2.85 were independently associated with an increased risk of severe postoperative complications (all *P* < 0.05, Table 3). Bootstrapping with 1,000 resamples confirmed the direction of these associations, albeit with wider 95% confidence intervals (CAR POD 3 ≥2.85: bootstrap OR 3.98, 95% CI 1.18–12.45), reflecting the limited number of severe events. Sensitivity analyses excluding patients with extreme CAR values (*n* = 4) or those undergoing combined procedures did not materially alter the main findings (Supplementary Table 1).

**Table 3:** Multivariable logistic regression analysis of factors associated with severe postoperative complications after radical gastrectomy in GC patients.

Factors	β	SE	Wald χ^2^	OR (95% CI)	*P*
Incision type	1.640	0.575	8.196	5.152 (1.678–15.820)	0.005
Operation duration	1.105	0.520	4.532	3.018 (1.092–8.340)	0.032
CRP POD3 ≥98.5 mg/L	0.541	0.115	21.592	1.720 (1.365–2.178)	< 0.001
CAR POD3 ≥2.85	1.236	0.546	6.248	4.287 (1.385–9.681)	< 0.001

### Predictive value of POD 3 CAR for severe postoperative complications after radical gastrectomy in GC patients

**Figure 2:**
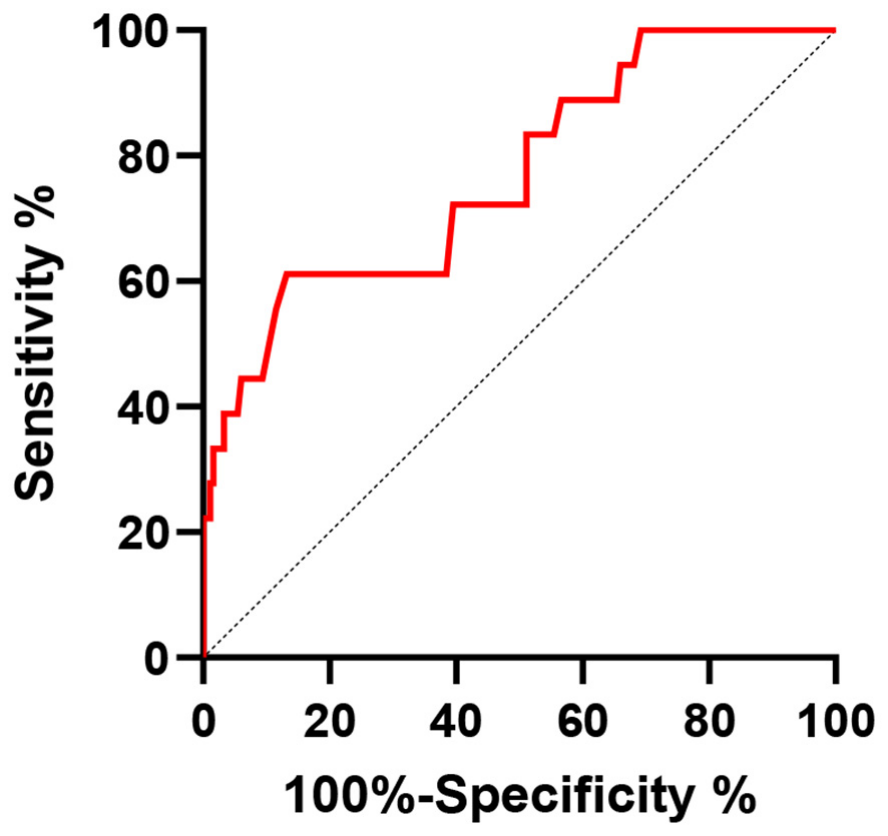
ROC curve of POD 3 CAR for predicting severe postoperative complications after radical gastrectomy in GC patients.

Based on the CAR data on POD 3, an ROC analysis model was established to evaluate the predictive value of CAR for severe postoperative complications after radical gastrectomy in GC patients. The ROC analysis revealed that the optimal threshold for CAR on POD 3 was 2.85. The area under the curve (AUC) for POD 3 CAR in predicting severe postoperative complications was 0.769, with a sensitivity of 0.745 and a specificity of 0.740, and a Youden index of 0.482 (Figure 2).

### Postoperative complication rates and hospital stay between the high CAR group and the low CAR group

Following the established critical CAR value, patients were categorized into the high CAR group and the low CAR group. The incidence rates of both mild and severe complications were significantly higher in the high CAR group compared to the low CAR group (31.58% vs. 8.06%, *P* < 0.001 for mild complications; 18.42% vs. 3.23%, *P* < 0.001 for severe complications). Additionally, patients in the high CAR group presented a longer postoperative hospital stay (*P* = 0.011, as shown in Table 4).

**Table 4:** Postoperative complication rates and hospital stay between the high CAR group and the low CAR group.

Item	CAR < 2.85 (*n* = 124)	CAR ≥2.85 (*n* = 76)	χ^2^	*P*
Clavien-dindo grade I or II complications	10 (8.06)	24 (31.58)	18.464	< 0.001
Clavien-dindo grade III or above complications	4 (3.23)	14 (18.42)	13.284	< 0.001
Postoperative hospital stay (days)	9.52 ± 1.57	10.23 ± 2.36	2.554	0.011

## Discussion

In this retrospective investigation, we assessed the prognostic utility of postoperative CAR for severe complications after radical gastrectomy in GC patients. Our findings suggest that CAR measured on POD 3 is associated with major postoperative morbidity, specifically complications classified as Clavien-Dindo grade III or higher; however, this association should be interpreted cautiously in light of the limited number of severe events and the exploratory nature of the present analysis.

The main finding of the current research was the strong link between an elevated POD 3 CAR (≥2.85) and a markedly increased risk of both mild and severe complications. Patients in the high CAR group demonstrated higher incidences of complications (31.58% vs. 8.06% for mild; 18.42% vs. 3.23% for severe) and a prolonged postoperative hospital stay. In the multivariable model, a POD 3 CAR ≥2.85, alongside an open surgical approach, prolonged operation time (≥270 min), and elevated POD 3 CRP (≥98.5 mg/L), was associated with severe postoperative complications. Nevertheless, because only 18 severe events were observed, the stability of this model may be limited, and the possibility of overfitting cannot be excluded. In addition, because CAR is derived from CRP and albumin, the simultaneous inclusion of CRP and CAR in the same multivariable model may introduce collinearity and should therefore be interpreted with caution. The ROC analysis showed moderate discriminatory ability of POD 3 CAR in this cohort (AUC=0.769). However, because this performance was not internally or externally validated, it should be considered exploratory rather than definitive evidence of clinical applicability. Consistently, Paliogiannis et al. suggested that CAR on POD 4 is an independent predictive marker for anastomotic leakage death in colorectal cancer patients who underwent elective surgery (13). Xu et al. indicated that CAR on POD 3 is closely linked to the occurrence of postoperative complications in colorectal surgery (14).

The predictive strength of CAR likely stems from its integration of two pivotal postoperative pathophysiological states: systemic inflammation and nutritional decline. CRP belongs to a well-established acute-phase protein that rises rapidly in response to surgical trauma and underlying infectious or inflammatory processes (15). An exaggerated or prolonged inflammatory response is detrimental to tissue healing and immune homeostasis, predisposing patients to infections, anastomotic leakage, and other complications (16). ALB, conversely, is a negative acute-phase reactant and a key indicator of nutritional status and hepatic synthetic function (17). However, postoperative albumin levels may also be influenced by perioperative fluid shifts, hemodilution, blood loss, and other non-inflammatory physiological disturbances, which means that the prognostic signal captured by CAR may not be entirely specific to inflammatory burden alone. Its postoperative decline reflects the catabolic state and nutritional stress induced by major surgery (18). Therefore, CAR effectively captures the dual burden of heightened inflammation and impaired nutritional reserve (19). Our results align with previous studies in other malignancies (20), reinforcing the concept that composite biomarkers reflecting the interplay between inflammation and nutrition often possess superior prognostic value compared to single parameters.

Notably, while preoperative CAR has been linked to long-term survival in GC, our study highlights the distinct importance of its postoperative trajectory. The lack of significant differences in preoperative and POD 1 values between groups underscores that the early postoperative inflammatory-nutritional shift, rather than the baseline state, is critical for complication development. Nevertheless, because proximal, distal, and total gastrectomy differ in operative extent and physiological stress, procedural heterogeneity may still have influenced postoperative inflammatory responses, even though resection type was not statistically significant in the univariate analysis. This finding directs clinical attention to the immediate postoperative period for early risk stratification and closer clinical surveillance.

The potential clinical implications of our study should be interpreted cautiously. POD 3 CAR is an easily obtainable and low-cost metric that may help identify patients at increased risk of major postoperative morbidity. However, because postoperative day 3 may coincide with the early clinical evolution of some severe complications, CAR at this time point is more appropriately interpreted as a marker for early postoperative risk stratification and clinical alert rather than a strictly preclinical predictor. Nevertheless, given the retrospective design, small number of severe events, and lack of validation, CAR should not be regarded as a stand-alone decision-making tool at present. Rather, it may be considered a hypothesis-generating marker that warrants further evaluation in prospective studies and in combination with other clinical variables.

Several limitations of our study must be acknowledged. First, its retrospective single-center design may introduce selection bias and limit generalizability. Second, the number of patients with severe complications was relatively small (*n* = 18), which may reduce the stability of the multivariable model and increase the risk of overfitting. Third, although internal validation via bootstrapping was performed to assess model stability, no external validation was conducted; therefore, the reported ROC performance of POD 3 CAR should be regarded as exploratory and requires confirmation in independent cohorts. Fourth, because CAR is calculated from CRP and albumin, the concurrent inclusion of CRP and CAR in the same multivariable model may have introduced collinearity, which was not formally assessed in the present study. Fifth, although all patients underwent radical gastrectomy with D2 lymph node dissection, variation in surgical procedures, including proximal, distal, and total gastrectomy as well as open vs. laparoscopic approaches, may have influenced postoperative inflammatory responses and complication patterns. Sixth, the use of Clavien-Dindo grade III or higher as a composite endpoint is clinically meaningful for capturing major postoperative morbidity, but it does not allow robust evaluation of the association between POD 3 CAR and individual complication subtypes. Finally, we did not analyze dynamic changes in CAR beyond POD 3, and the observational design cannot establish causality or elucidate the precise biological mechanisms linking a high CAR to postoperative complications. Future prospective multicenter studies with larger cohorts, dedicated validation strategies, and more comprehensive data exploration and sensitivity analyses are needed to confirm the robustness and clinical value of our findings.

## Conclusion

In conclusion, an elevated CAR on POD 3 was associated with severe complications following radical gastrectomy for GC in this retrospective cohort. As a composite marker reflecting postoperative inflammation and nutritional status, CAR may have potential value for early risk stratification. However, because of the limited number of severe events, possible model overfitting, the absence of external validation, and potential collinearity with CRP, these findings should be interpreted cautiously. While internal validation supported the general direction of the association, the wide confidence intervals highlight the need for confirmation in larger prospective multicenter studies before routine clinical implementation.

## Data Availability

The original contributions presented in the study are included in the article/Supplementary material, further inquiries can be directed to the corresponding authors.
