# Supplementary material for: Predictive value of postoperative C-reactive protein-to-albumin ratio for severe complications following radical gastrectomy in gastric cancer patients
Source: Front Med (Lausanne). 2026 Apr 30;13:1789300. doi: 10.3389/fmed.2026.1789300 (PMC13171757; doi:10.3389/fmed.2026.1789300)
Supplement: Supplementary file 1 [file Table_1.docx]

**Supplementary Table 1 Internal validation and sensitivity analyses for factors associated with severe postoperative complications**

| **Factor** | **Original OR (95% CI)** | **Bootstrap OR (95% CI)*** | **Sensitivity Analysis** (OR, 95% CI)** |
| --- | --- | --- | --- |
| Open incision | 5.15 (1.68-15.82) | 4.89 (1.52-14.96) | 4.72 (1.48-15.04) |
| Operation time ≥270 min | 3.02 (1.09-8.34) | 2.88 (0.98-8.52) | 2.79 (0.95-8.21) |
| CRP POD3 ≥98.5 mg/L | 1.72 (1.37-2.18) | 1.68 (1.32-2.14) | 1.65 (1.30-2.09) |
| CAR POD3 ≥2.85 | 4.29 (1.39-9.68) | 3.98 (1.18-12.45) | 3.85 (1.10-11.90) |

Notes: *Bootstrap based on 1000 resamples.
**Sensitivity analysis excluding patients with extreme CAR values (n=4) and those with major protocol deviations (n=3). CI, confidence interval; OR, odds ratio.
